# Three-dimensional broadband acoustic illusion cloak for sound-hard boundaries of curved geometry

**DOI:** 10.1038/srep36936

**Published:** 2016-11-11

**Authors:** Weiwei Kan, Bin Liang, Ruiqi Li, Xue Jiang, Xin-ye Zou, Lei-lei Yin, Jianchun Cheng

**Affiliations:** 1 Collaborative Innovation Center of Advanced Microstructures and Key Laboratory of Modern Acoustics, MOE, Institute of Acoustics, Department of Physics, Nanjing University, Nanjing 210093, P. R. China; 2 School of Sciences, Nanjing University of Science and Technology, Nanjing 210094, P. R. China; 3 Imaging Technology Group, Beckman Institute, University of Illinois at Urbana-Champaign, Urbana, Illinois 61801, USA

## Abstract

Acoustic illusion cloaks that create illusion effects by changing the scattered wave have many potential applications in a variety of scenarios. However, the experimental realization of generating three-dimensional (3D) acoustic illusions under detection of broadband signals still remains challenging despite the paramount importance for practical applications. Here we report the design and experimental demonstration of a 3D broadband cloak that can effectively manipulate the scattered field to generate the desired illusion effect near curved boundaries. The designed cloak simply comprises positive-index anisotropic materials, with parameters completely independent of either the cloaked object or the boundary. With the ability of manipulating the scattered field in 3D space and flexibility of applying to arbitrary geometries, our method may take a major step toward the real world application of acoustic cloaks and offer the possibilities of building advanced acoustic devices with versatile functionalities.

Invisibility cloak, with the capability of making scattering objects undetectable from the EM signals^1^,^2^,^3^,^4^,^5^,^6^,^7^ or acoustical signals^8^,^9^,^10^,^11^, has attracted rapidly growing attentions during the last decade. Among various methods developed for designing invisibility cloaks, the technique of coordinate transformation is a commonly used as well as powerful one^2^,^11^,^12^,^13^,^14^. By coordinately transforming the physical fields into an illusion where the space is filled with only background medium, the required material parameters for such device can be obtained. The parameters are often inhomogeneous, anisotropic, or of extreme values that do not exist in nature, until recently the advances in metamaterials make such parameters realizable^5^,^6^,^7^,^8^,^9^,^10^,^11^,^12^,^13^,^14^,^15^,^16^,^17^,^18^,^19^,^20^,^21^. Although numerous schemes have been proposed to design acoustic cloaks with practically feasible parameters^22^,^23^,^24^,^25^, only a few experiments have demonstrated the cloaking effect for acoustic wave so far. Zhang *et al*. and Popa *et al*. claim the demonstration of free-space cloaking in water and ground cloaking in air respectively. A three-dimensional (3D) axisymmetric cloak for a sphere in free-space^10^ is demonstrated by Sanchis *et al*., and a 3D ground cloak is proposed and fabricated by Zigoneanu^26^. More recently, a 3D invisibility cloak, allowing for concealing objects in open cavity with ellipsoidal section, is also devised and characterized^27^.

The existing cloaks can generally be divided into two categories: cloaks in free space^8^,^10^,^11^,^14^,^26^ and cloaks for hiding objects near reflecting boundaries^5^,^7^,^9^,^28^,^29^,^30^. For free-space cloaks, the assumption of an infinite free space necessarily brings difficulties or restrictions in the realization that often requires extreme parameters either negative^4^ or very inhomogeneous^2^,^14^, which usually limits the cloaks narrowband or unidirectional^10^,^11^,^26^. Although the ground cloaks can yield invisibility effect within broad bands, they only works near a totally flat boundary. Notice that the concept of ground cloak originates from the field of electromagnetics where it is more accurate to assume that the boundary is flat due to the relatively short wavelength of light or microwave. For airborne acoustic waves with wavelength of macroscopic dimensions, however, it would become less important to make the ideal-flat-boundary assumption, leading to the necessity to consider a feasible scheme for realizing cloaking effect near boundaries with arbitrary geometry. Besides, compared with invisibility cloak that only cancels the scattered field generated by the cloaked object^31^, an “illusion cloak”, by which the scattered field could be manipulated as if the object is transformed into some other object of one’s choice, would be more challenging. So far, the experimental demonstration for such cloaks is still few. A preliminary two-dimensional (2D) illusion cloak, capable of manipulating the acoustic field near boundaries of arbitrary curved geometry, is demonstrated by Kan^30^. The experimental realization of a 3D broadband boundary-independent acoustic illusion cloak should definitely take a major step toward the practical application of acoustic cloaking devices.

Here, we have designed, fabricated and characterized a 3D illusion cloak for camouflaging objects near curved sound-hard boundary on the basis of transformation acoustics. The designed cloak is composed by anisotropic subwavelength structures with effective parameters independent of either the cloaked object or the boundary. The device can work in broadband due to the absence of resonance element. The good performance of the fabricated design is characterized via measurements on the acoustic scattering from a cloak which is chosen, as a particular example, to make a round table near an axisymmetric bowl-shaped surface transformed into a bare surface. This 3D acoustic cloak with simple design and easy fabrication constitutes a further significant step toward the real world application of cloaking and offer new possibilities of versatile manipulation on acoustic waves.

## Results

**Figure 1:**
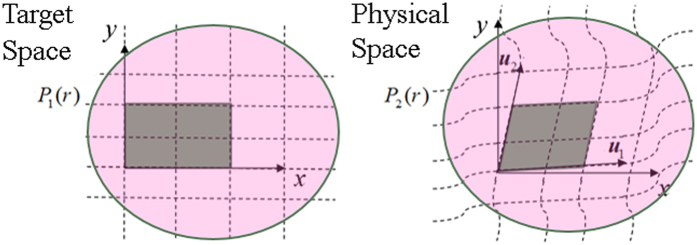
Schematic illustration of the coordinate transformation technique.

Suppose there are two different acoustic systems, the target system and the physical system, as shown in Fig. 1. The material distributions (marked by green) in the region (pink) surrounded by the elliptic circle are different, but out of the elliptic circle, everything is the same including the sound source, i.e., the boundary conditions of these two systems are the same. It is expectable that the acoustic fields for the two cases will be different, but when the material distribution in the physical system is properly designed, it is still possible to find a coordinate transformation *r* → *r* ′, (suppose ***r*** = ***xi*** + ***yj*** + ***zk*** is described with the Cartesian coordinate, and *r* ′ = *q*_1_***u***_1_ +  *q*_2_***u***_2_ + *q*_3_***u***_3_, where ***u***_1_, ***u***_2_ and ***u***_3_ are respective unit vectors), which makes the corresponding acoustic field *P*_2_(***r***) take the form of *P*_1_(***r*** ′) in the new coordinate system, without breaking the form of the wave equation. As *P*_1_(***r***) and *P*_2_(***r*** ′) take same mathematical form and have the same value on the boundary, the acoustic fields outside the green elliptic circle in the two cases will be kept the same under the excitation of arbitrary sound source, i.e., the two systems would appear the same under outside detections. Therefore, by choosing one of the systems as the predesigned target illusion, and using the other physical system to mimic the behavior of acoustic waves in the target space, the illusion effect can be achieved.

Define the metric factors in the new coordinate as


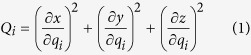


and the matrix


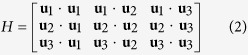


If the target system is already known, with the acoustic field *P*_1_(***r***) and material distribution written as *ρ*_i_ and *k*_i_, According to the geometric relationships between *P*_1_(***r***) and *P*_2_(***r***), i.e., the transformation ***r*** → ***r*** ′, the material distribution in physical space for *P*_2_(***r***) can be calculated as


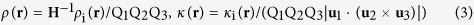


**Figure 2:**
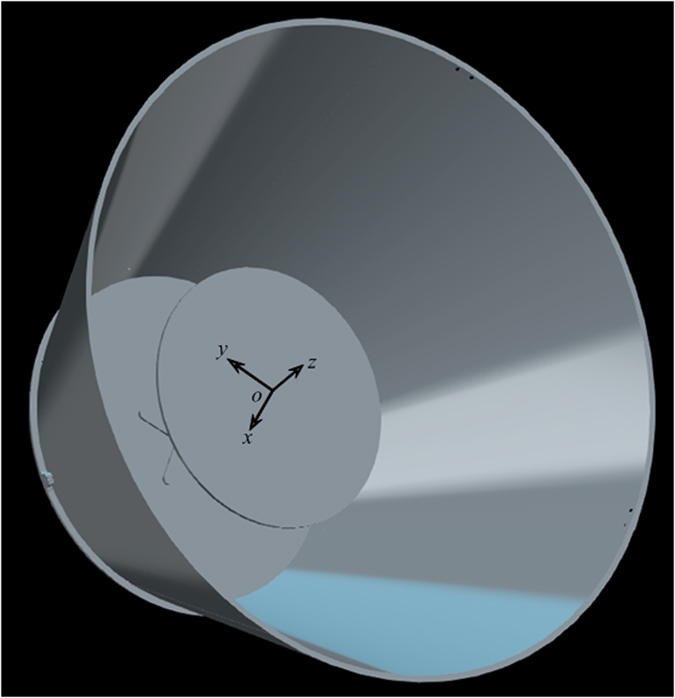
Schematic representation of the 3D object to be cloaked, chosen as a small round table placed in the bowl-like space.

The proposed illusion cloak can be obtained based on these formulae. For simplicity while without losing generality, we will demonstrate the proposed scheme by choosing a particular example as shown in Fig. 2. A small round table with a diameter of 12 cm and a height of 7 cm is placed near a curved sound-hard surface that forms a space with bowl-like shape (15 cm depth). Note that both the object and the boundary have axisymmetric configurations, which is only for facilitating the fabrication of experimental sample as well as improving the precision of measurement. It should be stressed that the validation of our scheme is actually independent of the shape of either the object or the boundary.

**Figure 3:**
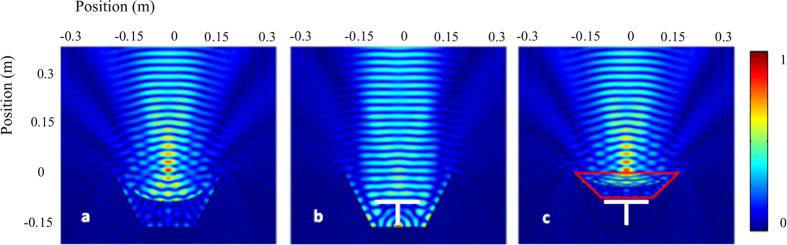
Numerical demonstrations in the symmetric plane of the structure, showing the effectiveness of the illusion cloak. Acoustic pressure amplitude fields (**a**) in the target illusion space, (**b**) disturbed by the small round table and the bowl-like sound hard boundary, (**c**) for the table covered by the cloak. The white object is the table and the proposed cloak is placed within the regions surrounded by red lines.

Our aim is to transform the whole system into a concave mirror under detection of acoustic signals by using a well-devised cloak covered onto the table, as shown in Fig. 3a. The fundamental design idea is acoustically squeezing the illusion space near the surface along one direction (the direction along the *z* axis in Fig. 2, i.e., the direction along the height of the table) into the area for inserting the cloak. By comparing the field in the illusion space and the cloaking area, the coordinate transformation should be specified by *q*_1_ = *x, q*_2_ = *y* and *q*_3_ = *z*/*n*_*c*_, where *n*_c_ take a value of 2.13 in this work.

The required parameters of the illusion cloak are calculated as *r*_*z*_ = 0.47*r*_*i*_ (***r***) (the effective mass density along z axis), *r*_*x,y*_ = 2.13*r*_*i*_ (***r***) (the effective mass density along = 0 plane), *k* = 0.47*k*_*i*_ (***r***) (the effective bulk modulus), where *r*_i_ and *k*_i_ are the material distributions in the illusion space as shown in Fig. 3a. The performance of such illusion cloak is numerically demonstrated in the symmetric plane (*y* = 0 plane) of the structure, as shown in Fig. 3. For all of the cases, a Gaussian beam is radiated from the upmost boundary and the frequency is 8 kHz. In Fig. 3a, the beam is reflected by the concave mirror and focused to the middle region, while in Fig. 3b, the beam is scattered by the combination of the table and the sound hard boundary. The difference between the acoustic fields shown in Fig. 3a and Fig. 3b is obvious. In Fig. 3c, with the reference system covered by the properly designed illusion cloak, the field in the physical space is successfully manipulated to restore the illusion field as shown in Fig. 3a.

**Figure 4:**
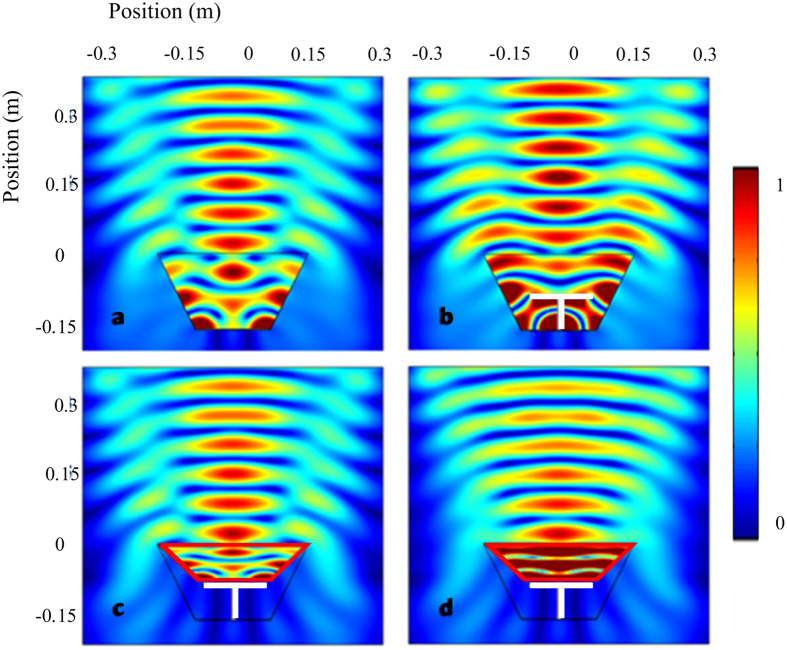
Numerical demonstrations in the symmetric plane of the structure, showing the effectiveness of the designed cloak. Acoustic pressure amplitude fields (**a**) near the bowl-like sound hard boundary, (**b**) disturbed by the small round table, (**c**) for the table covered by the ideal cloak, (**d**) for the table covered by the reduced cloak. The white object is the table and the proposed cloak is placed within the regions surrounded by red lines.

For the simplicity of characterization, we choose the target illusion system as the sound hard surface filled with the background medium (air in this work), then the illusion cloak can also be regarded as an invisibility cloak for the table. The material distribution in the illusion space in this case would be *r*_*i*_ (***r***) =  *r*_0_ and *k*_*i*_(***r***)=*k*_0_, and ideal values for the required parameters of the anisotropic cloak should be 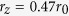, 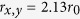, 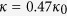, where 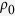 and 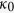 are the mass density and bulk modulus of air respectively. The performance of such cloak is numerically demonstrated in the symmetric plane (*y* = 0 plane) of the structure, as shown in Fig. 4.

The 3.43 kHz wave is radiated from the upmost boundary and scattered by the structures. In Fig. 4a, there is nothing in the space surrounded by the bowl-like surface. Hence the plotted field is the pressure amplitude pattern for the total field (superposition of the incident wave field and the scattered wave field) near the bowl-like sound hard surface. In Fig. 4b, the round table is inserted and affects the original field pattern obviously. Position shifts of the fringes due to superposition of the incident wave and scattered wave can be clearly observed. After covering the system with the devised cloak, the field pattern can be restored perfectly into the illusion one (see Fig. 4a,c) for the bare hard boundary. Because one component of the required effective density and the required bulk modulus is smaller than corresponding parameters of air, which suggests the metamaterial has to contain at least one basic material that is less dense and rigid than the background fluid. However, it is difficult to find such a material. Then all material parameters are scaled in the same direction^9^ and become 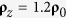, 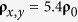, 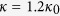, for easy fabrication of the conceived device. Under this condition, the impedance of the effective media is modified, while the phase information is conserved. Therefore, there is some reflection at the boundary of the cloak and the background media, which do not exist for the ideal condition. But the cloak with reduced parameters mimics acoustically the ideal cloak quite well. Such phenomenon can be observed in Fig. 4d. Although the total acoustic field pattern is not strictly the same with that in Fig. 4a and c, it agrees better with Fig. 4a when compared with Fig. 4b. In Supplementary Materials, the influence of impedance mismatch is qualified by comparing the difference of the resulting field in the far field for different systems. Furthermore, the effectiveness of the cloak and the influence of impedance mismatch for all incident angles are also checked in the Supplementary Materials.

**Figure 5:**
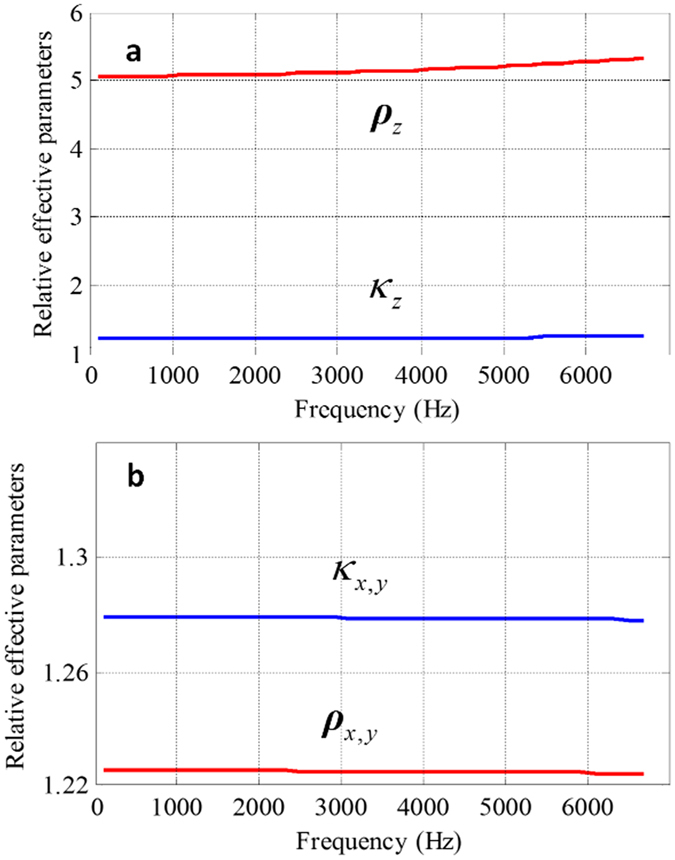
The effective parameters of the anisotropic structure. (**a,b**) The retrieved effective parameters of this structure as a function of frequency when the wave normal incidents to the sheet (**a**) and goes along the sheet (**b**). The red line is the normalized effective mass density and the blue line is the normalized bulk modulus.

For implementation of such parameters by metamaterials, we employed the subwavelength anisotropic structure that can be easily made by drilling square array of holes in metal sheets. The geometric parameters determine the anisotropy and the effective acoustic parameters. By studying the normal incidence plane wave reflection and transmission of the structure, the geometry parameters are properly designed for the required effective mass density and bulk modulus. The thickness of the sheet is 1 mm and the diameter of the hole is 1.6 mm. Both of the distances between metal sheets and the neighboring holes are chosen as 5 mm, which is 1/20 of the wavelength for frequency of 3430 Hz and sufficiently small to ensure the validation of the effective medium approximation. The retrieved effective parameters of this structure are calculated with the method proposed in ref. 21 and shown in Fig. 5a,b as functions of frequency. The red line is the normalized effective mass density and the blue line is the normalized bulk modulus. It can be observed from Fig. 5a that in the low frequency region, from 0 Hz to approximately 4 kHz, the effective parameters nearly remains constant, which implies the resulting device is able to work in this broad band region. And the band width can be broadened further by simply scaling down the unit cell sizes. Unlike the local resonant structures with working band depending on the dimension of the unit cells, the lower bound of the working frequency of this designed anisotropic structure is irrelevant of the unit cells, while the upper bound can be promoted by scaling down the cell size provided that the viscosity effect is negligible. While the frequency increases to above 4 kHz, the structure will scatter acoustic waves obviously, and behave more like a sonic crystal, as a result, the effective parameters no longer remain constant. The 3D unit cell has advantage over the 2D unit cell used in ref. 30, where the upper bound of working frequency is about 2 kHz with the same cell dimension. For the 3D structure, the diagonalized mass density tensor should have three components. But due to the symmetry of this structure, the components of density tensor 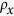 and 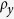 are the same. Hence we have only two independent components as we plotted in Fig. 5a,b respectively. Figure 5a is for the case where the wave normally incidents onto the sheet, whereas the effective density *ρ*_*z*_ is much larger than *ρ*_*x,y*_ in Fig. 5b. Such a structure will increase the momentum in the direction vertical to the sheets, while has trivial influence in the direction along the sheets. As the value of *k*_z_ and *k*_*x*,y_ comes very close, the bulk modulus of such structure can be practically regarded as isotropic.

**Figure 6:**
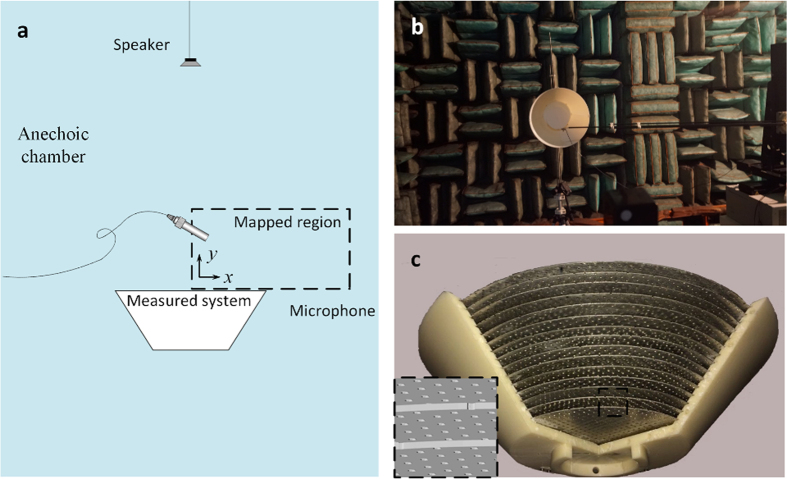
Experimental setup. (**a**) The schematic of the experimental setups used to map the acoustic field near the cloak. The mapped region is indicated together with the sound source. (**b,c**) The photo of (**b**) the whole measuring system and (**c**) the prototype of cloak with one side of the shell is taken off to show the interior structure. Inset: zoom-in view of the perforated sheets.

The perforated metal sheets are obtained by punching holes periodically in the thin metal sheets, and then the whole structure is fabricated by assembling the sheets with a properly designed shell molded by a commercial 3D printer (Stratasys Dimension Elite, 0.177 mm in precision). Figure 6a is the schematic of experimental setup, along with the photos of the setup (Fig. 6b) and prototype (Fig. 6c). In the measurement, we put the loudspeaker 1 m away from the structures to mimic the plane wave field. Considering the fact that the wavelength of the measuring signal is about only 0.1 m, this can be regarded a considerably accurate approximation. Measurements were done in an anechoic chamber. The acoustic pressure amplitude fields corresponding to the bare bowl, the bowl with the round table, this reference system covered by the cloak, are measured in the mapped region. For the axisymmetric configurations of both the object and the boundary, the scanned region is selected in the *xz* plane (only the right side in Fig. 6a) such that the illusion effect could be clearly identified. Although the proposed scheme applies to arbitrary scenarios, and under the detection of any acoustic signals, we only take the bowl and table system for facilitating the experiment.

**Figure 7:**
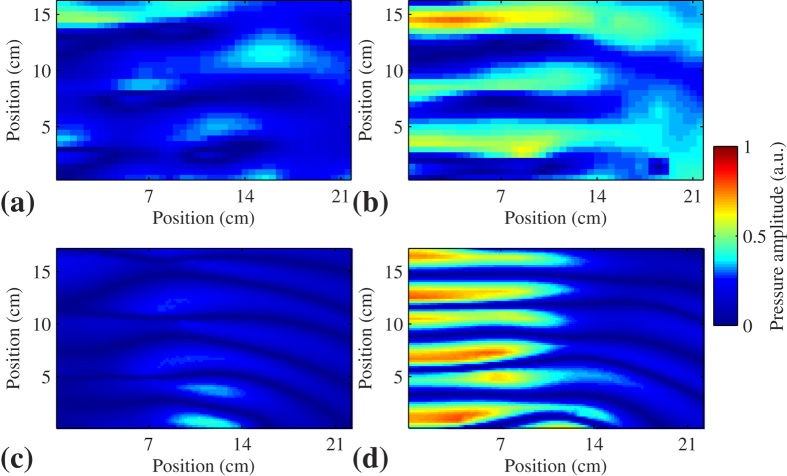
Experimental results. Experimental results for the cloak with its corresponding numerical result. The field of FD in the y = 0 plane when the target is chosen as (**a**) the table with the cloak, and (**b**) the table without the cloak placed in the bowl-like space. **(c,d**) Corresponding numerical results.

Because the dimension of the reference system (around 10 cm) is only of the same order of the detecting wavelength, the scattered wave by the measured system is not significantly altered. As shown in Fig. 4a,b, the contrast between the scattered fields generated by cloaked and uncloaked objects is not remarkable. For unambiguously evaluating the performance of the designed cloak, we use the parameter of field disparity (FD) defined as 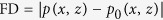 with 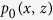 and 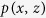 being the pressure amplitudes at 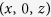 generated by the illusion object and by the reference system with or without the illusion cloak. FD gives a quantitative estimation on how well the cloak mimics acoustically the target illusion effect, and a perfect illusion is achieved when FD vanishes. Vanishing of FD means the characterized system perfectly mimics the target system under detecting acoustic signals. Figure 7 illustrates the comparison of the FD obtained via numerical simulation and experimental measurement for two cases where the reference system is covered by the cloak [Fig. 7a] and the reference system is not covered by the cloak [Fig. 7b] respectively. A good agreement is observed between the numerical prediction and the experimental result. It is apparent that the acoustic scattered field without the cloak is significantly different from that in the illusion space, as shown by the large fluctuations in Fig. 7b–d. Contrarily, both the numerical and experimental results of the FDs are negligible (see Fig. 7a,c) when covered with the cloak, indicating that the illusion of the bare bowl is created by the cloak. The broadband performance of the device is also characterized experimentally (see Supplementary material). Compared to the previous cloaking devices, this scheme can be easily extended to design other related cloaking devices by choosing a more complex and practical target space, instead of the illusion with only the background media. Under the detection of outside signals, the object will be acoustically transformed in to the freely-designed illusion by the proposed 3D illusion cloak. The required parameters will be similarly devised and the fabricating complexity only depends on the complexity of the acoustical parameter distribution of the chosen illusion space.

The sound source in the experiment is adopted as a 5 cm diameter loudspeaker, with a specific directional pattern. As the measured system and mapped area are in the far field region and well aligned with the sound source, the wave field was verified as a good plane wave field in the mapped area before setting up the measured system. Besides, the sound attenuation due to viscous effect was not considered in this work, although the attenuation, especially in the cloaking medium, may bring some influences to the result and will be addressed in the future research. These conditions may lead to the difference between patterns of FDs in simulation and experiment, but do not obviously impair the performance of the cloak.

The proposed scheme for designing illusion cloak is a general one which features broadband and omnidirectional performance, and allows the parameters of cloaking media to be independent of either the cloaked objects or the geometry of the boundaries. Besides, there is no restriction on the size of the cloaked objects in theory. But it is necessary to note that some factors should be taken in to consideration for proper design of the cloak. For example, it is still difficult to cloak a large object with such devices of limited size, and the main difficulty lies in constructing high relative refractive index medium without strong impedance mismatch between the medium and air. Although the problem of impedance mismatch can be avoided for cloaking in water, to find a high relative refractive index material in water is more difficult. It is possible to cloak any object from any detecting signals with such devices big enough, but the fabrication would become a mission impossible when the dimension of the cloak is too big and the signal frequency is too high, regarding the dimensions of the unit cells should be much smaller than the wavelength in the background medium. Therefore, the resulting device will be a balance between the performance of the device and the difficulty in its fabrication.

## Discussion

We reported the experimental demonstration of 3D acoustic illusion cloak for objects near arbitrary curved surfaces by exploiting the technique of transformation acoustics. The cloak medium is only composed by homogeneous and anisotropic metamaterial, of which the parameters are realized by subwavelength structures with no demanding of resonating elements for negative values. Moreover, the parameters of the cloak shell are independent of the properties of either the original object or cloaked region, which brings significant convenience for the design of such devices. The fabricated 3D cloak is verified to be able to function in a broad bandwidth and for all incident angles of detecting signals, and transform a sound hard bowl with an inserted scattering object into a bare bowl. Compared with the 2D device that only perform in the waveguides in laboratories, this proposed 3D cloak has the flexibility of applying to arbitrary geometries and the potential of generating other free designed acoustic illusion by detection of various acoustic signals, which will significantly facilitate the practical application for such devices in real world.

## Methods

### Numerical Simulation

The numerical simulation in Figs 3 and 4 is performed with general PDE module of COMSOL Multiphysics^®^ (the commercial software package based on finite-element method). The background medium is air with its density 1.21 kg/m^3^ and sound speed 343 m/s. The solid materials are reasonably assumed to be rigid as compared to air.

### Acoustic Measurement

The experiment is carried out inside an anechoic chamber, the setups and acoustic field mapping area is illustrated in Fig. 7. A 5 cm-diameter sound speaker is used to excite the approximate plane wave field around the structures with a sinusoidal signal. Considering the difficulty in fabricating the cloak devices with subwavelength structure at high frequency region, the measurement is carried out under a relatively low driving frequency of 3.43 kHz to guarantee the precision of the structural parameters of sample. The sound pressure is measured by 0.25-inch-diameter Brüel & Kjær Type-4961 microphones. The microphone is fixed on to a linear stage with graphite shaft and can move in the mapping region with high accuracy. The recording and analysis equipment contain a Brüel & Kjær PULSE 3160-A-042 multichannel analyzer and a desktop computer with Brüel & Kjær PULSE software LabShop version 13.5.10.

## Additional Information

**How to cite this article**: Kan, W. *et al*. Three-dimensional broadband acoustic illusion cloak for sound-hard boundaries of curved geometry. *Sci. Rep.* **6**, 36936; doi: 10.1038/srep36936 (2016).

**Publisher’s note:** Springer Nature remains neutral with regard to jurisdictional claims in published maps and institutional affiliations.

## Supplementary Material

Supplementary Information
