# Supplementary material for: Three-dimensional broadband acoustic illusion cloak for sound-hard boundaries of curved geometry
Source: Sci Rep. 2016 Nov 11;6:36936. doi: 10.1038/srep36936 (PMC5105145; doi:10.1038/srep36936)
Supplement: Supplementary Information [file srep36936-s1.pdf]

Supplementary Material to the article “Three-dimensional broadband acoustic illusion cloak for sound-hard boundaries of curved geometry”

Weiwei Kan, Bin Liang, Ruiqi Li, Xue Jiang, Xinye Zou, Lei-lei Yin and Jianchun Cheng.

The simulation results for oblique plane wave incidence

We have quantified the performance of the 3D acoustic cloak by inspecting the total pressure field with oblique acoustic wave incidence via numerical simulations. The numerical results are plotted in Figs. R1 and R2 in which two particular cases are considered with incident angle chosen as $\pi / 9$ and $2\pi / 9$ respectively. Since the scattering object is placed inside the bowl-like space with sound hard boundary, the scattered wave by the object will be weakened as the incident angle increase, which makes it more difficult to directly compare the pressure fields for cases with and without the cloak. For evaluating the performance of the cloak more clearly when the incident angle becomes larger, we introduce the parameter of field disparity (FD) defined as $\mathrm{FD} = \left| p(x,z) - p_0(x,z) \right|$ with $p_0(x,z)$ and $p(x,z)$ being the pressure amplitudes at $(x,0,z)$ in the target space and in the mapped area for cloaked or uncloaked systems respectively. FD gives a quantitative estimation on the difference between the two mapped amplitude fields. Vanishing of FD means the scattered field generated by the designed device mimics perfectly that of the target system. From the numerical results of FDs illustrated in Fig. R2, it can be observed that results of the FDs are negligible when covered with the designed cloak, indicating that the cloak also works for oblique incident waves and give rise to the desired acoustic illusion

that the sound hard bowl with the inserted object acoustically "becomes" a bare bowl. We have also compared the performances of the ideal cloak and the reduced one with scaled parameters. As demonstrated by the numerical results, both the ideal and reduced cloaks are quite effective when illuminated by obliquely-incident acoustic waves, except for slight scattering from the reduced cloak due to the impedance mismatch caused by scaling the parameters.

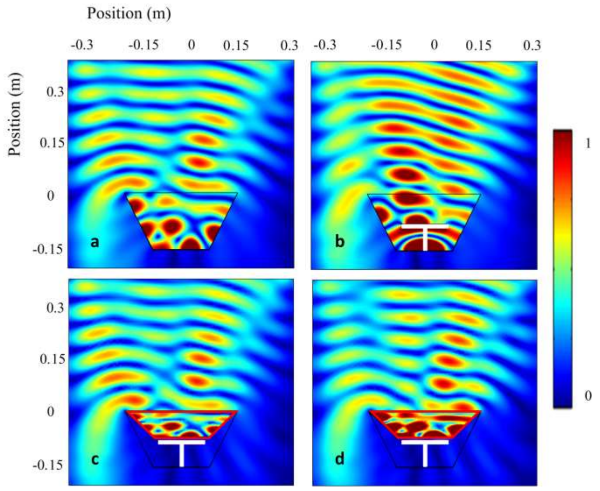

Figure S1 | Numerical demonstrations for the case of oblique acoustic wave incidence with incident angle of $\pi / 9$. (a-d) 2D cross section views of the acoustic pressure amplitude fields (**a**) near the bowl-like sound hard boundary, (**b**) disturbed by the small round table, (**c**) for the table covered by the ideal cloak, (**d**) for the table covered by the reduced cloak. The white object is the table and the proposed cloak is placed within the regions surrounded by red lines.

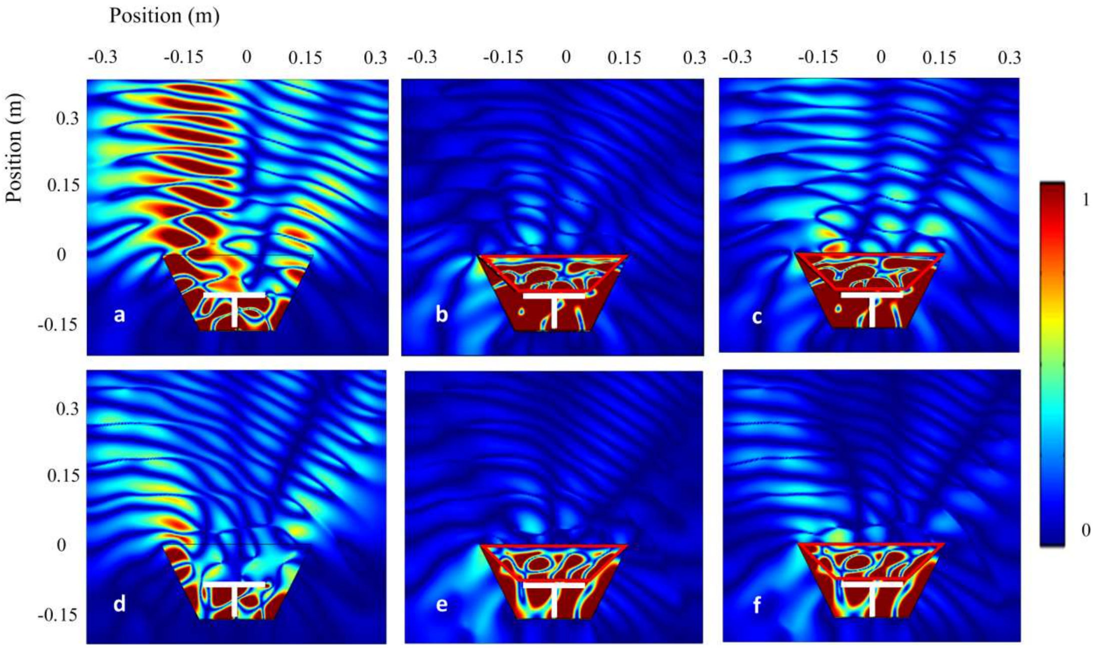

Figure S2 | Numerical results of FD. The FD with incident angle of $\pi/9$ when the target is chosen as (**a**) the table without the cloak, (**b**) the table with the ideal cloak and (**c**) the table covered by reduced cloak; The FD with incident angle of $2\pi/9$ when the target is chosen as (**d**) the table without the cloak, (**e**) the table with the ideal cloak and (**f**) the table covered by reduced cloak. The white object is the table and the proposed cloak is placed within the regions surrounded by red lines.

Qualification of the difference between the performance of the reduced device and the ideal device

The final effective parameters for the fabricated cloak in the manuscript are obtained by scaling the ideal parameters, as illustrated in Ref. 1. Here are some numerical results to illustrate the difference between the ideal and the reduced transformation spaces. And some discussions about the influence of the impedance mismatch on the reflection at the boundary between the cloak and the background medium. In the reduced transformation space, by scaling the mass density and bulk modulus of the cloaking medium obtained in the ideal space, the refractive index is conserved but the

impedance of the medium is changed. However, in the reduced transformation space, the validity of the designed cloak will not be affected by this change of impedance, as will be demonstrated by the numerical results given below. Because the sound hard wall exist behind the cloak in the scheme, it is difficult to qualify the reflection at the boundary between the cloak and the background media directly. For quantitatively evaluating the reflection at the surface of the cloak as well as the effect of impedance mismatch caused by the enlarged parameters, we have defined the disparity in the far field (DIFF) for different systems as $r\left|p(x,z)-p_0(x,z)\right|$ at $r\to\infty$, where $r=\sqrt{x^2+z^2}$, and still with the scattered field from the empty bowl-like sound hard boundary chosen as the reference. In the ideal transformation space, there exist no reflection at the boundary between the cloak and the background media, and the scattered field from the empty bowl-like sound hard boundary would be perfectly restored, leading DIFF to approach zero at all angles. While in the reduced transformation space, the reflection at the boundary of the cloak will lead to a nonzero value of DIFF. The result of DIFF in the reduced transformation space is given in Fig. S3 as function of the incident angle. It turns out that the cloak with reduced parameters mimics acoustically the ideal cloak quite well, characterized by the remarkable suppression of DIFF (blue) in comparison to the no-cloak case (red). Although the DIFF for reduced cloak does not reach zero as in ideal case due to slight impedance mismatch, the cloak still significantly suppress DIFF, esp., the three main lobes around the backscattering direction.

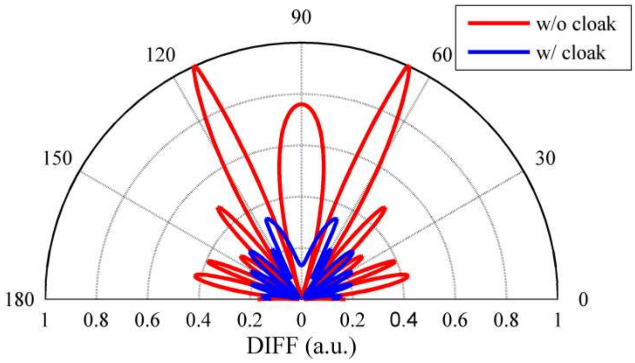

Figure S3 | The result of DIFF as a function of incident angle, when the reference object is covered with no cloak (red) and reduced cloak (blue).

Broadband performance of the device

Experiments are carried out in order to address the issue of broadband functionality. We define a parameter as

$$\gamma(\omega)=\frac{1}{T}\int\frac{\int\left|\mathrm{Re}(p_{\mathrm{t}}-p_i)\right|ds}{\int\left(\left|p_{\mathrm{t}}\right|+\left|p_i\right|\right)ds}dt\,,$$

where p_{t} is the pressure field measured for cases with or without the cloak, and p_{i} is the pressure field for the target illusion object. The time averaging is performed over the period $T=2\pi/\omega$. In this case the parameter γ quantitatively evaluates the difference between the measured field and the target illusion field. In this case the parameter γ quantitatively evaluates the performance of the cloak. $\gamma=0$ means the measured system perfectly mimics the behavior of the illusion system under the detection of acoustic signals. We measured this parameter of different samples illuminated by an oblique incidence wave with an incident angle of 20° in the anechoic chamber to characterize the broadband functionality. Figure S4 shows the frequency dependence of γ obtained from the measurements (symbols) or from the

finite element simulations (continuous lines). The red (blue) data is the result of the case where target is chosen as the reference object without (with) the cloak. It is clear that the value of γ is notably reduced in the characterized frequency region, revealing the broadband functionality of the designed cloak.

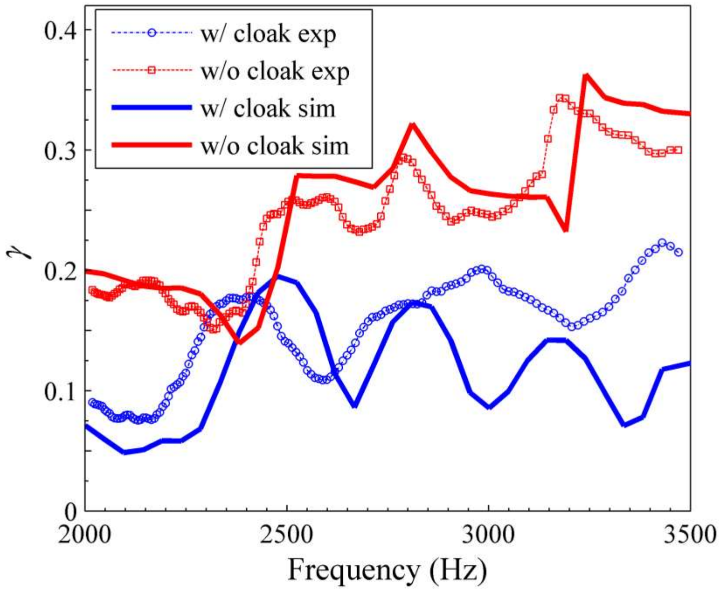

Figure S4 | Simulated (dashed lines) and measured (symbols) γ as functions of wavelength. Red (blue) data: target chosen as the reference object without (with) the cloak.

References:

[1] Popa, B.-I. & Cummer, S.A. Design and characterization of broadband acoustic composite metamaterials. Phys. Rev. B 80, 174303 (2009).
